# Microinvasive glaucoma surgery: a review and classification of implant‐dependent procedures and techniques

**DOI:** 10.1111/aos.14906

**Published:** 2021-05-14

**Authors:** Joanna Jabłońska, Katarzyna Lewczuk, Joanna Konopińska, Zofia Mariak, Marek Rękas

**Affiliations:** ^1^ Department of Ophthalmology Military Institute of Medicine Warsaw Poland; ^2^ Department of Ophthalmology Medical University of Bialystok Białystok Poland

**Keywords:** glaucoma, implants, microinvasive surgery, MIGS

## Abstract

The aim of this article is to discuss how physiology and anatomical background affect the effectiveness of implant‐dependent microinvasive glaucoma surgery (MIGS). First, we provide a micro view of aqueous outflow and tissue behaviour. Second, we review studies exploring the mechanisms of the pressure‐lowering effect of MIGS, as well as tissue behaviour during aqueous flow and tissue motion. We also describe and classify microinvasive surgical procedures and the most important types of implants, as well as their mechanisms of action, implantation techniques and efficacy. Further, we summarize the indications and surgical results presented in recent studies, providing an evidence‐based update on novel and emerging MIGS techniques for the treatment of open‐angle glaucoma. These data can help surgeons to personalize the management of glaucoma and to choose the best MIGS option for individual glaucoma patients.

## Introduction

Trabeculectomy has been the ‘glaucoma surgery of choice’ for years. However, due to the risk of vision‐threatening complications, as well as a decrease in postoperative effectiveness with time, a constant search for safer and more effective surgical techniques has been underway (Soltau et al. 2000; Gedde et al. 2012). For over 10 years, intensive research on minimally invasive methods of glaucoma surgery, termed microinvasive glaucoma surgery (MIGS), has been conducted (SooHoo et al, 2014). Ahmed and Saheb proposed that MIGS treatments should be characterized by the following five properties (Saheb & Ahmed 2012): an *ab interno* approach through a clear corneal incision that spares the conjunctiva; use of procedures that minimize trauma to the target tissue; an intraocular pressure (IOP)‐lowering efficacy that justifies the approach; a high safety profile that avoids serious complications compared to other glaucoma surgeries; and rapid recovery with minimal impact on the patient's quality of life (Caprioli et al. 2015). Subsequently, in 2014, the American Glaucoma Society and the US Food and Drug Administration (FDA) characterized MIGS as the implantation of a surgical device intended to lower IOP via an outflow mechanism, with either an *ab interno* or *ab externo* approach associated with very little or no scleral dissection (Francis et al. 2011).

Microinvasive glaucoma surgery (MIGS) is intended to achieve lower IOP in patients with glaucoma with less surgical time and, ideally, to have a medication‐sparing effect. The surgical techniques involved are based on the physiological aspects of aqueous humour (AH) flow in the eye; furthermore, the choice between various anatomical sites in the eye globe influences the range of the corresponding IOP‐lowering effects. To date, a reduction in IOP is the only proven method to slow the progression of visual field loss (Gedde et al. 2012; Saheb & Ahmed 2012; SooHoo et al, 2014). Because of increasing life expectancy, patients live longer with glaucoma (Brandão & Grieshaber 2013) and are at a risk of glaucoma progression over a longer period. Therefore, it is essential to operate on glaucoma at an early stage and to lower the IOP intensively from the beginning. However, the role of MIGS in the glaucoma treatment algorithm has yet to be fully determined. In this paper, we review the characteristics and clinical outcomes of the most frequently used implants in MIGS. We also discuss their advantages and biases, along with factors that should be considered in future studies.

For this review, we used PubMed to conduct an online search of literature published from 2015 to 2020, using keywords appropriate to this topic. For an overall description of MIGS implants, we included studies with the following keywords: trabecular micro‐bypass stent, iStent Supra^®^ (Glaukos, San Clemente, CA, USA), Schlemm's canal scaffold, Hydrus^®^ (Ivantis, Irvine, CA, USA), suprachoroidal microstent, CyPass^®^ Micro‐Stent (Alcon, Fort Worth, TX, USA), XEN^®^ Gel Stent (Allergan, Irvine, CA, USA), and PRESERFLO^®^ MicroShunt (Santen, Osaka, Japan). To assess clinical outcomes, we included randomized clinical trials (RCTs) comparing MIGS with trabeculectomy or other therapies, observational studies and other methodologies. Research on MIGS as a solo surgery or in conjunction with cataract extraction was also examined.

## Physiology of Aqueous Drainage

In order to better explain the mechanism of action of the implants, we present details of the anatomy and physiology of Schlemm's canal (SC) and the mechanisms of AH outflow.

Aqueous humour (AH) is drained from the eye via two physiological pathways (Achache 2001; Morrison & Pollack 2003). The conventional path begins at the level of the irido‐corneal trabecular meshwork (TM) and accounts for approximately 83–96% of drainage (Achache 2001). From the anterior chamber, the AH moves through the TM to SC and the intrascleral connecting channels, which lead to the intrascleral venous plexus, aqueous vessels and venous vessels of the suprascleral space. Aqueous vessels begin as collector channels (CC) in the exterior wall of SC and can be seen on the surface of the eye in the corneal limbus (Morrison & Pollack 2003).

Drainage can also occur by an unconventional suprachoroidal pathway that does not begin in the trabeculum (Morrison & Pollack 2003; Tamm 2009). Small amounts of AH can pass through the cornea and vitreous and thus through the retina and optic disc. However, unconventional drainage mainly takes place through the anterior part of the choroid, also referred to as the suprachoroidal pathway (Pederson et al. 1977; Morrison & Pollack 2003). Drainage via this path takes place through the base of the ciliary muscle (where the AH is produced), which does not have an endothelial barrier to the anterior chamber (Pederson et al. 1977). Use of this drainage pathway decreases with age, from 30% to 35% in young persons (25–30 years old) to 3% in individuals aged over 60 years (Pederson et al. 1977). The reason for this phenomenon is assumed to be the stiffening of tissues, which increases with age (Wang et al. 2017).

## Drainage Path Structure and Outflow Resistance

Aqueous humour (AH) flows out of the anterior chamber as a mass stream regulated by a pressure gradient (Pederson et al. 1977) and fills SC. The pressure in SC must be lower than that in the anterior chamber to permit AH flow. This reduction in SC pressure simultaneously requires a one‐way mechanism to prevent backflow of the AH into SC from the episcleral veins. The pressure in these veins is normally lower than that at the entrance of the CCs, which in turn must be lower than that in SC to permit AH flow. The AH flows through the drainage pathways at an average rate of 2.0 *µ*L/min (Johnson & Kamm 1983). In healthy human eyes, outflow facility has a value of 0.40 *µ*L x min/mm Hg at 10 mm Hg (Brubaker 1975), but this value decreases with age (Gaasterland et al. 1978). From a physiological perspective, the trabeculum, particularly the interior wall of SC, and the TM near the CC are the main sources of resistance to AH outflow; the remaining resistance likely comes from the exterior wall and surrounding tissues (Johnson & Kamm 1983; Rosenquist et al. 1989). This area is called the juxtacanalicular space and is assumed to be the primary site of IOP regulation (Goel et al. 2010). Thus, elevated IOP in glaucoma is caused by an increase in outflow resistance in the AH drainage pathway rather than by an increase in AH production (Achache 2001). Moreover, this outflow resistance is not constant—it is a function of IOP and rises as IOP rises (Brubaker 2003).

## Schlemm's Canal

Schlemm's Canal (SC) was named in honour of the German anatomist, Friedrich Schlemm, who, in 1830, discovered the canal in the anterior chamber angle through which the AH is taken into the bloodstream (Dvorak‐Theobald 1955; Mansouri & Shaarawy 2015). Schlemm's canal (SC) drains AH from the trabeculum into the suprascleral and conjunctival veins via CCs. It is a circuitous channel 36–40‐mm long and 190–370‐*µ*m wide (Achache 2001). Its interior wall consists of a continuous monolayer of endothelium (Ethier 2002), in which the endocellular route of AH flow is found, represented by giant vacuoles and pores (Achache 2001). Giant vacuoles are potential spaces between the extracellular matrix and the inner wall cells of SC (Ethier 2002). They form dynamically and respond instantaneously to changes in IOP (Epstein & Rohen 1991; Dautriche et al. 2015), and their quantity and size increase as IOP increases. The majority of giant vacuoles are found near CC outlets (Parc et al. 2000), which suggests that a greater pressure gradient is present there due to the increased aqueous flow (Ethier 2002). Pores are structures in the inner wall ranging from 0.6 to 3 *µ*m in size (Ethier 2002; Braakman et al. 2014) and are responsible for approximately 10% of the resistance to AH drainage (Alvarado et al. 2004). They can be found in the walls of giant vacuoles, but may be functionally unrelated to them (Tamm 2009). Pores form the main pathway of aqueous flow through the inner wall of SC.

The interior diameter of SC changes in response to IOP fluctuations (Johnstone & Grant 1973; Johnstone 1979) but is too large to generate significant resistance in the outflow path (Achache 2001). When the IOP rises, the TM expands towards the lumen of the canal, causing it to narrow. At high IOPs, parts of the canal's lumen close, increasing the probability that its walls will collapse and increase resistance in drainage routes (Battista et al. 2008); the canal does not collapse under the influence of physiological increases in IOP in healthy eyes (Ten Hulzen & Johnson 1996). Extensive collapse of the canal only occurs at pressures of 40 mm Hg or higher, with the exception of points where the septa are located (which do not collapse) (Van Buskirk 1982).

**Fig. 1:**
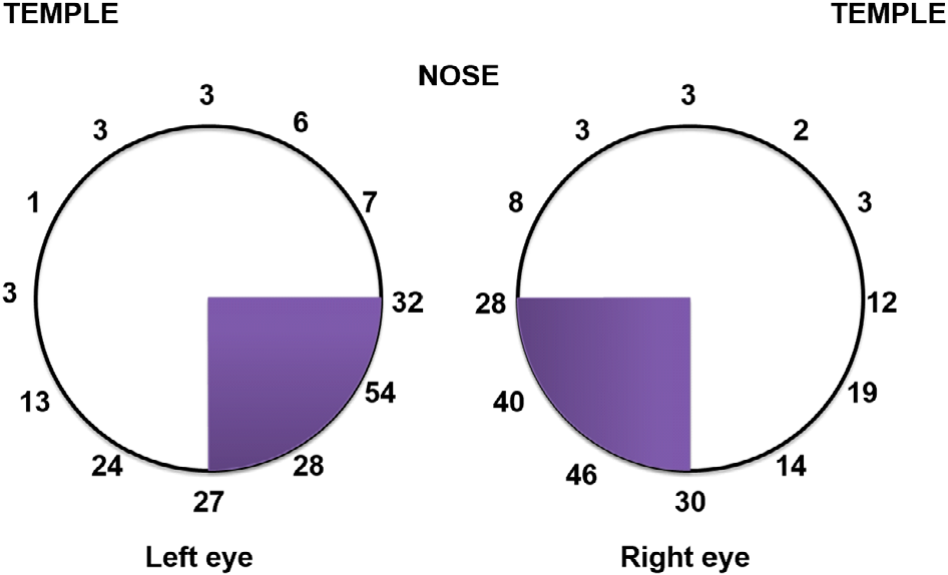
Aqueous vein density.

The AH flows out of SC through one of the 30 CCs and aqueous veins and then to the system of suprascleral veins (Rosenquist et al, 1989), ophthalmic veins and the general circulation (Morrison & Pollack 2003). Aqueous veins are approximately 1 mm in length and 50 *µ*m in diameter (Rosenquist et al. 1989) (Fig. 1). According to Hagen–Poiseuille's law, the resistance of aqueous veins should be insignificant if they are not collapsed or compressed (Dietlein et al. 2000). Therefore, distal aqueous drainage routes do not appear to play a significant role in generating outflow resistance (Johnson & Kamm 1983).

## Scientific Background of the Outflow Mechanism

Intraoperative provocative gonioscopy and channelography were evaluated in a study conducted by Grieshaber et al. (2009); their multinomial regression model results revealed that higher IOP levels are correlated with lower blood reflux rates independently of age. Provocative gonioscopy, during which blood reflux into SC is observed, is the simplest method of assessing the conventional drainage pathway. It also facilitates localization of an uncollapsed collector or aqueous vein (Johnstone et al. 2011), enabling the assessment of the blood reflux pattern. Provocative gonioscopy is performed intraoperatively in an eye with paracentesis‐induced hypotony (Grieshaber et al. 2010). Blood reflux is not provoked by a compression of the episcleral veins by the gonioscopic lens, but rather by the hypotony itself. Using this technique and an assessment of the amount of blood present in the angle, three filling patterns can be determined: no filling, incomplete filling and complete filling (Grieshaber et al. 2010). The technique also provides information related to SC patency and its connection to a patent distal system. A more precise assessment of the distribution of aqueous veins can be obtained through canalography (Video S1) and involves the direct injection of fluorescein into SC using a microcatheter during the canaloplasty procedure (Grieshaber et al. 2009). Episcleral outflow can then be assessed and graded according to the number of vessels that fill with dye in each quadrant. The most frequent point of implantation in SC surgery is the nasal quadrant, mainly because surgical access through a temporal corneal incision is straightforward. The nasal quadrant contains most of the CCs and aqueous veins (Fig. 1; Videos S2 and S3).

Zhou & Smedley (2005) introduced the trabeculum bypass theory, which proposes reducing resistance in this part of the drainage route. They hypothesized the presence of two types of bypasses, permitting either unidirectional or bidirectional flow, incorporated through boundary conditions for solving the equations and deriving the facility of outflow and the reduced IOP. According to their hypothesis, the amount of outflow increases by 13% and 26% in the presence of a unidirectional and bidirectional bypass, respectively. The circumferential flow is significant only in the immediate quadrant of the bypass. The authors also observed increased flow through SC only in the quadrant where the implant was applied. Intraocular pressure (IOP) reduction was dependent on the initial pressure (Zhou & Smedley 2005), and SC and CC dilation significantly lowered the IOP. With trabecular bypass alone, the elevated IOP in primary open‐angle glaucoma (POAG) is expected to drop to the mid‐to‐high teens (15–19 mm Hg). Intraocular pressure (IOP) can be further reduced by another 3–6 mm Hg with moderate SC and CC dilation; however, the circumferential length of the dilated SC affects the efficacy of IOP reduction. In theory, SC dilation using a trabecular bypass is analogous to a partial trabeculotomy in terms of IOP reduction. Within the scope of the concept of segmental flow, trabecular bypass operations may not reduce the IOP as much as do traditional procedures. This is likely because, in patients with POAG, the number of opened CC remains the same when IOP increases (as it does in healthy eyes), causing increased outflow resistance.

## Suprachoroidal Space

Prostaglandin analogues (latanoprost, travoprost) can increase suprachoroidal outflow and thus lower the IOP. Certain authors suggest that the suprachoroidal outflow route may undergo greater modification with the use of medications than the conventional route, as described above in the paragraph ‘Physiology of aqueous drainage’ (Toris 2010; Winkler & Fautsch 2014). This is also why attempts are made to use this path in MIGS. Emi et al. (1989) suggested that a negative pressure gradient of 3–4 mm Hg is generated between the suprachoroidal space and the anterior chamber, creating a potential driving force for AH outflow to the suprachoroidal space. The pressure difference between the anterior chamber and the posterior suprachoroidal space increases at higher IOP (Emi 1989).

**Fig. 2:**
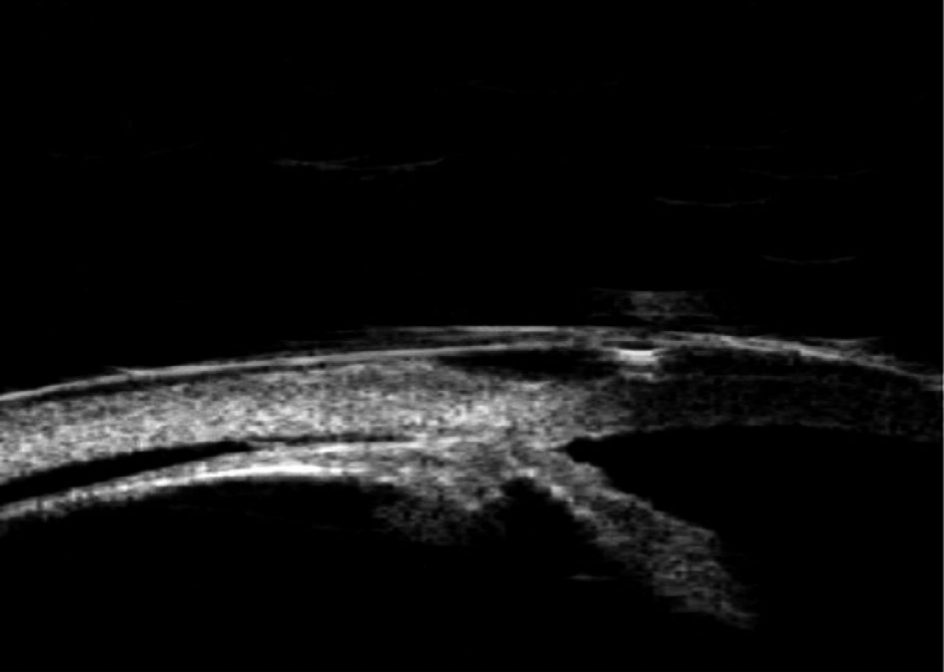
Cyclodialysis is visible in ultrasound biomicroscopy.

Cyclodialysis is the separation of the longitudinal muscle of the ciliary body from the scleral spur. Aqueous humour (AH)flows directly through this cleft into the suprachoroidal space and causes hypotony, a phenomenon described for the first time over a century ago (Fuchs 1900). Intentional surgical cyclodialysis has been used for the treatment of glaucoma and was first described by Heine in 1905. Surgical cyclodialysis is achieved by inserting a spatula between the sclera and choroid through a posterior scleral incision into the anterior chamber (Fuchs 1900). Ultrasound biomicroscopy image of cyclodialysis is shown in Fig. 2.

However, achieving controlled cyclodialysis for the purpose of therapeutic IOP reduction is difficult and attempts to surgically increase suprachoroidal outflow have had limited success due to various complications (Gentile et al. 1996; Ozdamar et al. 2003; Jordan et al. 2007; Razeghinejad & Spaeth 2011). Suprachoroidal devices may also be considered if trabecular stents fail or if the target pressure cannot be achieved using these stents. Depending on their mechanism of IOP‐lowering action, MIGS implants can be classified as those that improve conventional outflow, those that enhance unconventional outflow, and those that bypass conventional outflow (Saheb & Ahmed 2012; Brandão & Grieshaber 2013; Schmidt et al. 2013). In this review, we focus on six devices in these classifications:

- SC implants that correct conventional outflow
  ▪ iStent^®^
  ▪ Hydrus^®^
- Suprachoroidal space implants that strengthen outflow via the unconventional route
  ▪ CyPass^®^
  ▪ iStent Supra^®^
- Implants that bypass physiological aqueous drainage pathways
  ▪ XEN^®^ Gel Stent
  ▪ PRESERFLO^®^ MicroShunt

### The iStent®

The first‐generation iStent^®^ trabecular micro‐bypass stent (European Union CE certified & FDA approved, 2012) was designed to restore natural physiological outflow by creating a patent bypass to SC through the TM. It targets the increased resistance caused by the juxtacanalicular part of the TM, which is believed to represent the site of greatest resistance to AH outflow in patients with POAG (Johnson 2006). It has an ‘L’‐shaped structure with a snorkel‐shaped inlet on the short side (which sits in the anterior chamber) and an open half‐pipe lumen. At 1.0 mm in length and 0.33 mm in height, a snorkel length of 0.25 mm and a diameter of 120 *µ*m, it is the smallest device approved for use in humans. The convex side of the iStent^®^ sits against the inner wall of SC, with the open half‐pipe against the outer wall. Mathematical models of AH outflow project the size of the lumen to be more than adequate to accommodate the flow induced by the stent (Yuan et al. 2016). Separate orientations of the iStent are available for the right eye (OD) and for the left eye (OS). The iStent^®^ is inserted *ab interno* through a small temporal clear corneal incision and placed in SC at the lower nasal quadrant (Video S1). Implantation of the stent at this location allows the AH to bypass the obstructed TM and drain directly from the anterior chamber into SC; this also optimizes outflow in the lower nasal quadrant area, which has the highest concentration of CCs (Le & Saheb 2014) (Fig. 1.).

The iStent^®^ is manufactured using titanium, a commonly used medical implant material with proven biocompatibility. A heparin coating ensures wetting of the lumen for self‐priming. It is nonferromagnetic and thus safe for magnetic resonance (MR) imaging, although nonclinical testing has demonstrated that iStent^®^ models GTS100R and TS100L are MR‐conditional, that is, safe for use in specified MR environments under specified conditions (https://www.glaukos.com/en-uk/istent-inject-w-procedure/innovative-design/). Specifically, a patient with this device can be safely scanned in an MR system meeting the following conditions: static magnetic field of 3T or less, maximum spatial gradient magnetic field of 4000 gauss/cm (40 T/m), maximum MR system reported and a whole body averaged specific absorption rate of 4 W/kg (first level‐controlled operating mode; http://www.glaukos.com/istent/design). Several stents can be used to achieve a better hypotensive effect (Katz et al. 2015).

### The iStent Inject^®^

**Fig. 3:**
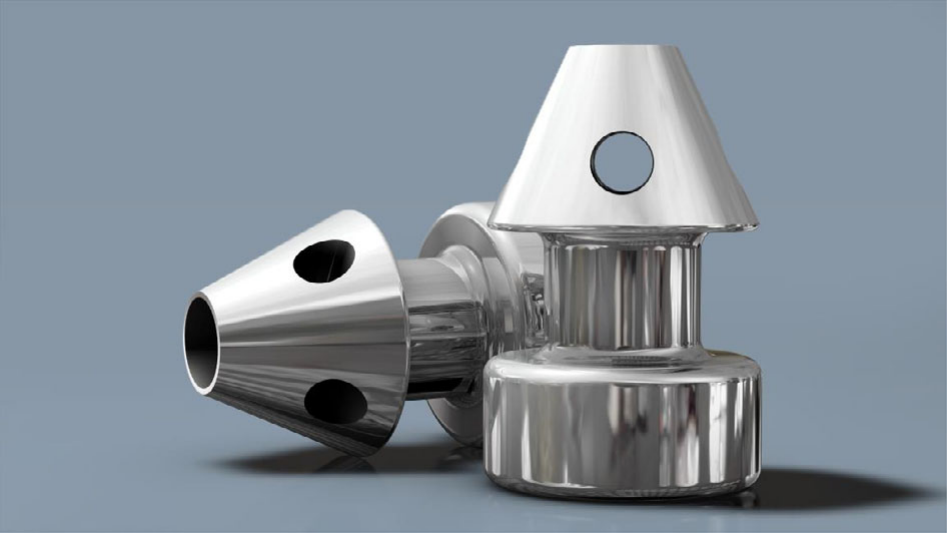
iStent^®^ Inject implant.

The iStent Inject^®^ is a second‐generation trabecular micro‐bypass stent recently introduced to provide additional IOP reduction. Similar to the first‐generation iStent, the iStent Inject^®^ is made of biocompatible, medical‐grade titanium. The system contains an injector preloaded with two heparin‐coated titanium stents, each with a central lumen and four side outlets to permit for multidirectional aqueous outflow. The implants are placed *ab interno* on two distinct areas of the TM into SC where AH subsequently flows into CCs. The two stents are able to tap into up to 6 clock hours (i.e. half of the total span of the angle) of the CCs. The placement of stents in two separate regions enables access of AH to more CCs, theoretically enabling a more pronounced decline in IOP (Salimi et al. 2019) (Fig. 3). Both generations of the iStent^®^ are contraindicated in eyes with primary‐ or secondary‐angle closure glaucoma, including neovascular glaucoma, as well as in patients with a retrobulbar tumour, thyroid eye disease, Sturge–Weber Syndrome or other type of condition that can cause elevated episcleral venous pressure. Gonioscopy should be performed prior to surgery to exclude peripheral anterior synechiae (PAS), rubeosis and other angle abnormalities or conditions that prohibit adequate angle visualization, which could lead to improper placement of the stent (Videos S2 and S3).

### The iStent Supra^®^

**Fig. 4:**
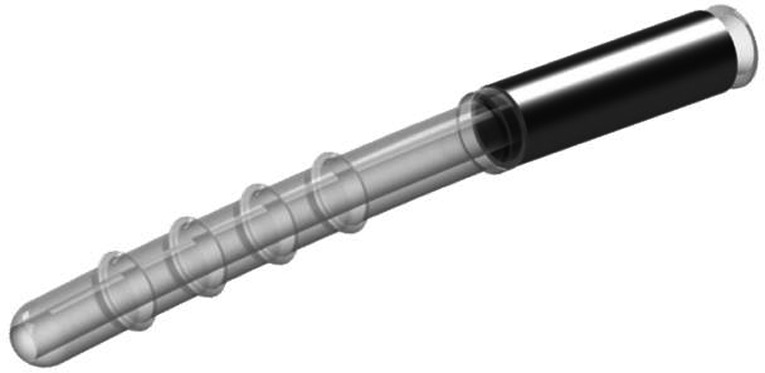
iStent^®^ Supra implant.

The iStent Supra^®^ is a third‐generation stent made from polyethersulfone and a coloured titanium sleeve (Fig. 4). It consists of a 4‐mm long tube with an opening at each end and is placed in the suprachoroidal space via *ab interno* access through a clear corneal incision. It is designed to create a patent lumen from the anterior chamber into the suprachoroidal space and has retention rings to provide stability at the implant location. The iStent Supra^®^ has not yet been commercially released and, thus, limited information is available at present.

**Fig. 5:**
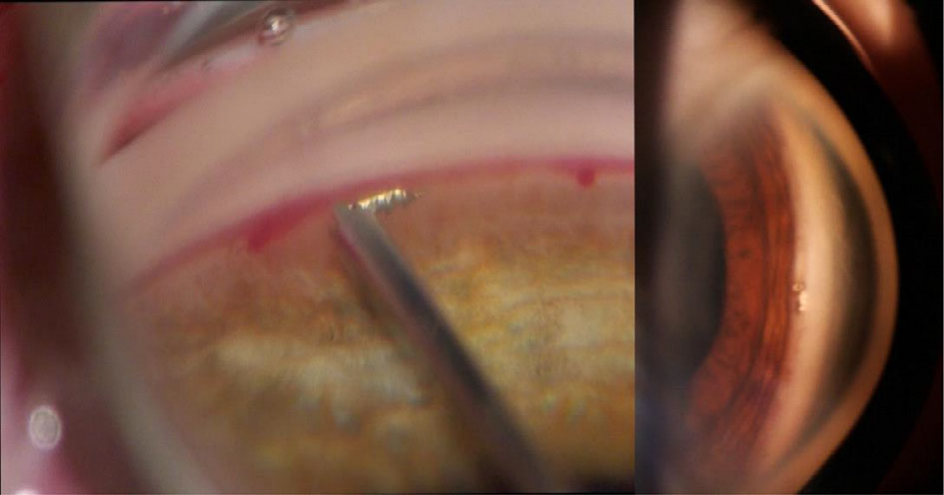
iStent^®^ implant inserted during gonioscopy.

The iStent^®^ first generation is currently the most widely used type of stent. The efficacy and safety of micro‐bypass by itself or in combination with phacoemulsification has been assessed in numerous studies (Fea 2010; Fernández‐Barrientos et al., 2010; Arriola‐Villalobos et al., 2012; Fea et al. 2014). Samuelson et al. (2011) presented the results of a study on iStent^®^ implantation with simultaneous cataract removal surgery (Fig. 5). The study involved a year‐long observation of 240 eyes randomly categorized into two groups: in the first group, patients underwent cataract phacoemulsification surgery with iStent^®^ implantation; only cataract removal was performed in the second group. In the iStent^®^ group, 72% of treatment eyes achieved an unmedicated IOP of ≤21 mm Hg at the 1‐year time point, compared to 50% in the control group. The safety profile was similar in both groups (Samuelson et al. 2011). Fea et al. (2015) conducted a trial of iStent^®^ versus phacoemulsification alone in a randomized setting, with results assessed up to 16 months, finding a statistically significant difference in final IOP between the groups. Ferguson et al. (2016) reported a 21% reduction in postoperative IOP at 24 months in a real‐world study of 350 American eyes that underwent iStent^®^ implantation in combination with cataract surgery, while Gallardo et al. (2016) reported a 31% reduction in IOP after 3 years in a mainly Hispanic population. Craven et al. (2012) reported a statistically significant therapeutic effect in an iStent^®^ group in their 2‐year observational study.

Studies have also confirmed beneficial effects of implanting multiple stents, and that multiple implantation may reduce the number of administered medications (Belovay et al. 2012). In a nonrandomized prospective case series study, Belovay et al. (2012) compared IOP among patients with two and three stents. The authors suggested that the observed dependence of pressure on the number of applied stents indicated that the surgeon can optimize the number of implanted stents according to each patient's target IOP. Additionally, a study involving patients with refractory glaucoma (after prior trabeculectomy) who received either two iStent^®^ trabecular micro‐bypass stents or one iStent Supra^®^ suprachoroidal stent for the treatment of refractory glaucoma, along with postoperative travoprost (Myers et al. 2018). At the 48 months, 97% of the eyes in the first group versus 98% of those in the second group achieved IOP ≤15 and ≤18 mm Hg, respectively, on one medication, indicating that both stents have similar IOP‐lowering effectiveness.

The most frequently described complications after iStent^®^ implantation are hyphema, transitory increase in IOP, corneal oedema, an obstructed stent, implantation‐related difficulties, entrapment of the vitreous, improper stent positioning and the necessity of repeating the procedure (Arriola‐Villalobos et al. 2012; Belovay et al. 2012; Craven et al. 2012; Ferguson et al. 2017; Pillunat et al. 2017; Esfandiari et al. 2019; Le et al. 2019).

### The Hydrus^®^ Microstent SC scaffold

**Fig. 6:**
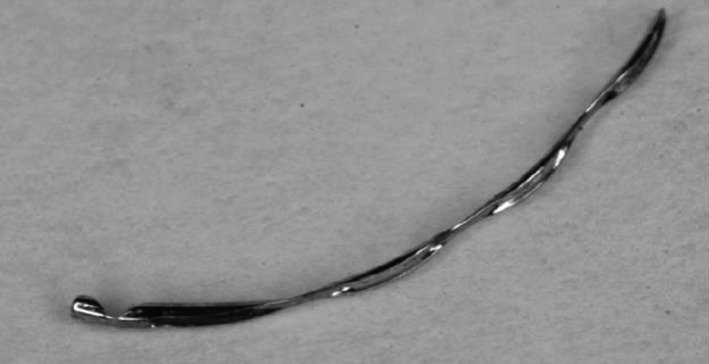
A Hydrus^®^ implant.

**Fig. 7:**
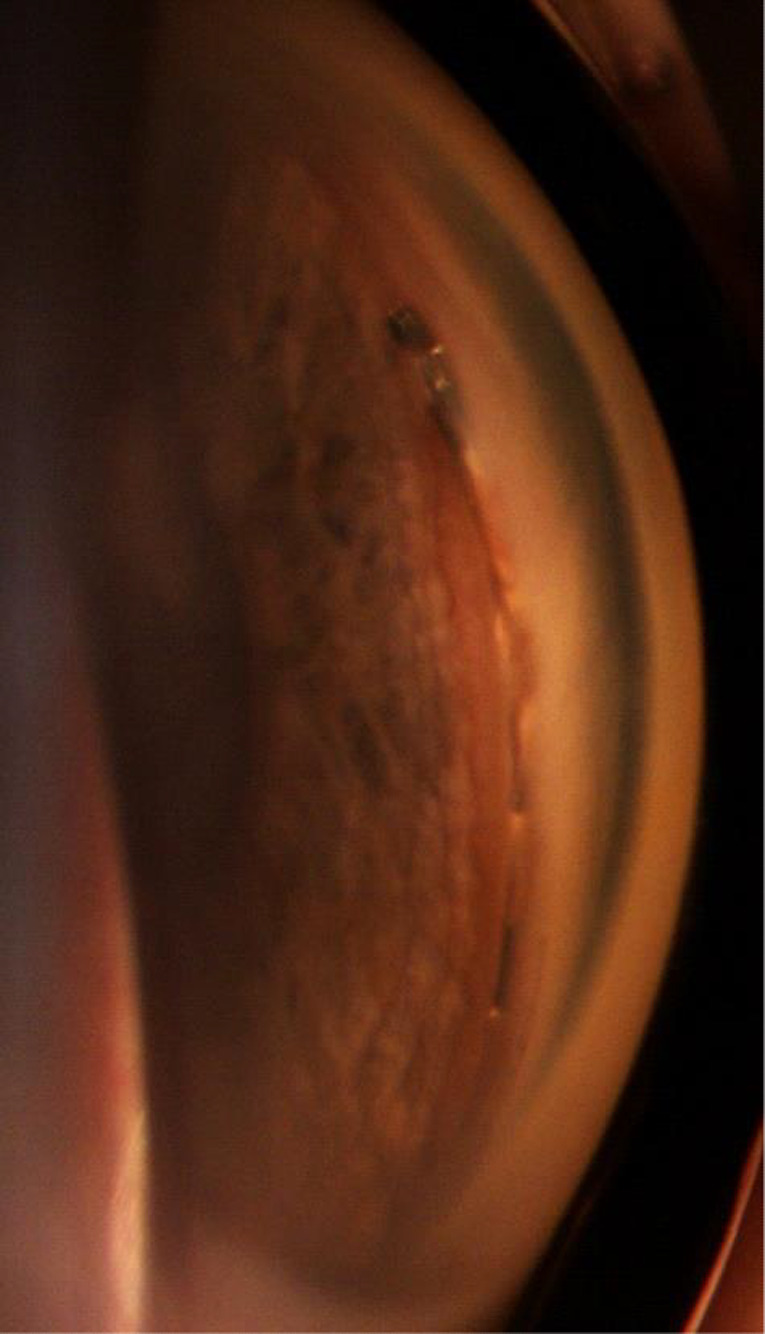
A Hydrus^®^ implant in gonioscopy.

The Hydrus^®^ Microstent SC scaffold is a CE‐certified SC scaffold that directly bypasses the TM to drain AH into SC. It is made from nitinol®, a nickel‐titanium alloy, is flexible, biocompatible, and contains three windows over its 8 mm length (Figs 6 and 7) (Mansouri & Shaarawy 2015). The implant is designed to enable increased outflow of AH from the anterior chamber into SC (Grierson et al. 2015) (Video S4). The Hydrus microstent dilates approximately one quadrant of SC (3 clock hours), and the most common site of implantation is the nasal quadrant. The proximal 1 mm inlet section of the microstent remains outside SC in the anterior chamber, ensuring direct inflow of the AH (Gulati et al. 2013). Studies conducted on eyeballs collected from human cadavers have shown significant improvement in outflow after implantation of the Hydrus microstent under different perfusion pressures (Camras et al. 2012). Saheb and Ahmed published data from a 6‐month observation of 28 eyes after phacoemulsification with Hydrus implantation and reported that the average initial IOP dropped from 29 to 15 mm Hg (Saheb & Ahmed 2012). Pfeiffer et al. (2015) showed a statistically significant greater reduction in IOP over a 2‐year period in a Hydrus plus cataract surgery group compared to a surgery alone group. No statistically significant differences in terms of safety were observed. In the COMPARED study, stand‐alone Hydrus implantation in POAG patients resulted in a higher surgical success rate and fewer medications compared with implantation of two iStents^®^, with the safety profiles being similar for the procedures. In that study, 47% of patients with Hydrus implants and 24% of those with the two iStents were medication‐free at 12 months. The percentage of eyes reaching ≤18 mm Hg without medications was greater in the Hydrus group (30% versus 9%), as was the percentage of eyes reaching a 20% or more reduction in IOP from the washed‐out baseli (40% versus 13%). The mean IOP for eyes without medications was 17.3 ± 3.3 mm Hg in the Hydrus group and 19.2 ± 2.4 mm Hg in the two iStents^®^ group (mean change = −8.2 mm Hg versus −5.1 mm Hg; difference in change, −3.1 mm Hg). Complications in both groups included subconjunctival haematoma, hyphema, PAS and IOP spikes of 10 mm Hg or more (Pfeiffer et al. 2015; Otarola et al. 2020).

### The XEN^®^ Gel Stent

**Fig. 8:**
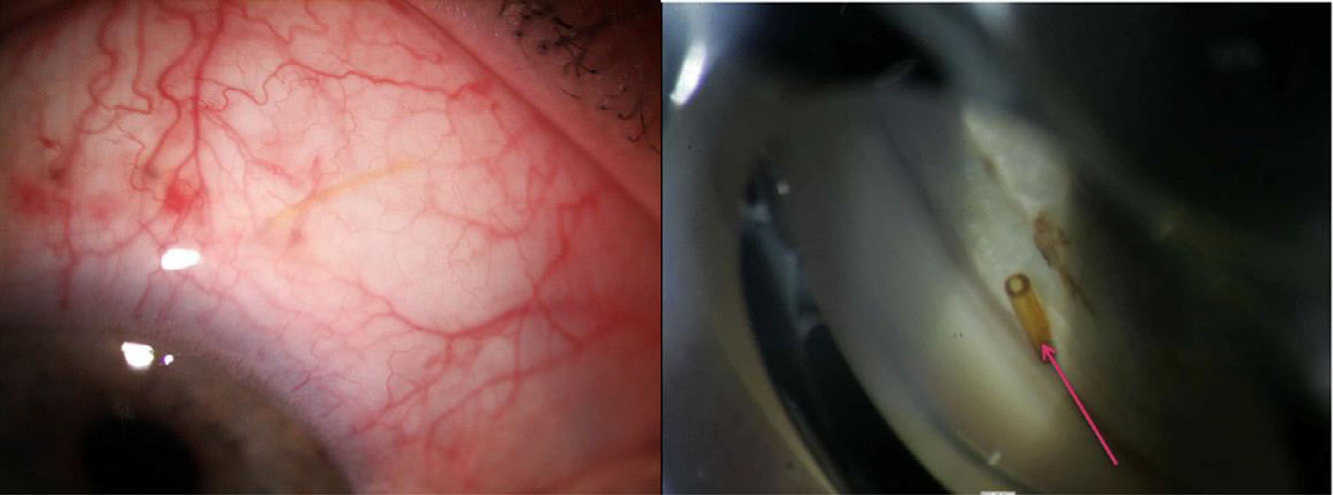
A XEN^®^ implant.

The XEN^®^ Gel Stent (CE certified in 2011): this category of subconjunctival MIGS stents was developed with the aim of improving the predictability and safety profile of bleb‐forming procedures (Do et al. 2020). The stents are FDA approved for use with cataract surgery and stand‐alone procedures (Lewis 2014). They are nonabsorbable implants made from a soft, cross‐linked collagen tube with a length of 6 mm, and interior lumen diameters of 140, 63 and 45 *µ*m in the Xen 140, Xen 63 and Xen 45 versions, respectively. Unlike silicone tube shunts, they do not incite significant inflammation or a foreign body tissue reaction, reducing the risk of fibrous proliferation, progressive inhibition of aqueous flow, and bleb failure (Lewis 2014). Their design is based on the Hagen–Poiseuille equation, with the pressure difference across the tube estimated based on its length and internal lumen diameter to avoid postoperative hypotony (Lewis 2014) (Fig. 8). The lowest AH resistance is offered by XEN^®^ 140. The XEN^®^ 63 lowers the AH resistance up to 2–3 mm Hg and XEN^®^ 45 retains 6–8 mm Hg of resistance for aqueous outflow, warranting the least risk of hypotony (Sheybani et al. 2015; Sheybani et al. 2016). The only model commercially available at the moment is the XEN^®^ 45. However, results from an early study of the XEN^®^ 63 were recently published (Lavin‐Dapena et al. 2020). XEN^®^ stents can be implanted by both *ab externo* and *ab interno* approaches. In the *ab interno* approach, the stent is delivered into the anterior chamber through an inferotemporal clear corneal incision made with a 27‐G sharp bevelled needle tip. The sharp tip is introduced at the TM and advanced through the sclera to exit approximately 2.5–3 mm posterior to the limbus into the subconjunctival space. The internal and external locations are verified, and the anterior chamber is irrigated to ensure flow and bleb formation (Green et al. 2018). The implant connects the anterior chamber to the subconjunctival space transsclerally (Fig. 8). The concept of the transscleral XEN^®^ implant is based on utilizing the outflow route produced by trabeculectomy and bypassing potential points of outflow resistance while conserving the conjunctiva. The bypassing of all potential points of outflow resistance together with the *ab interno* access eliminate the need to create a scleral flap and reduce the probability of the related complications that can accompany traditional antiglaucoma surgeries (Video S5).

Studies evaluating the stent's safety and efficacy in reducing IOP in patients with early‐stage, medium‐stage and advanced‐stage glaucoma are in progress. Initial reports indicate that the XEN^®^ implant enables effective IOP control and a reduction in the number of antiglaucoma medications administered (Lewis 2014), and has a favourable safety profile. Pérez‐Torregrosa et al. (2016) assessed the safety and effectiveness of phacoemulsification combined with XEN^®^ 45 implant surgery in 30 POAG eyes with concomitant cataract over a 12‐month observation period. The surgery was augmented with subconjunctival mitomycin C (MMC). The mean preoperative IOP of 21.2 ± 3.4 mm Hg decreased by 29% at 12 months, and the average number of medications decreased by 95%. Similarly, De Gregorio et al. (2018) implanted XEN^®^ 45 Gel Stents in 41 eyes in combination with phacoemulsification. Complete success, defined as a postoperative IOP ≥6 and ≤17 mm Hg without glaucoma medications, and qualified success, defined as a postoperative IOP ≥6 and ≤17 mm Hg with medication, were achieved in 80% and 98% of the cases, respectively. After 12 months, the mean postoperative IOP was 13.1 ± 2.4 mm Hg (mean IOP reduction of 42%) with a mean of 0.4 ± 0.8 medication classes (p < 0.05 for both IOP and medications).

Several authors have reported similar effectiveness of XEN^®^ implants in lowering IOP (Lewis 2014; Pérez‐Torregrosa et al. 2016; Galal et al. 2017; Smith et al. 2019). Laborda‐Guirao compared the effectiveness of combined XEN^®^ 45 with phacoemulsification surgery with solo procedure in a retrospective study of 80 eyes with 12 months of follow‐up (Laborda‐Guirao et al. 2020). They did not find any significant differences in success rates, number of IOP‐lowering medications or re‐operations between groups. The authors concluded that XEN^®^ implants alone or in conjunction with phacoemulsification are effective in treating patients with advanced POAG. Wagner et al. (2020) retrospectively compared the effectiveness of XEN^®^ to trabeculectomy in 171 eyes with refractory glaucoma. After 12 months of follow‐up, there were no statistically significant differences between the groups in the complete success rates (66% and 59% in the trabeculectomy and XEN^®^ groups, respectively) or in the ratio of needling and complication rates. Results from the longest follow‐up study for XEN^®^ 45 (36 months) were recently published for a cohort of 91 eyes (Gillmann et al. 2020a; Gillmann et al. 2020b). Complete success based on the criterion of IOP ≤15 mm Hg or a 20% reduction from baseline (medication free) was achieved in 29% of eyes at 3 years after surgery. There were no significant differences in efficacy between the XEN^®^ 45 procedure performed solo versus when combined with phacoemulsification. For XEN^®^ 63, the longest follow‐up as of this writing is 5 years, but the study population consisted only of 11 eyes (Lavin‐Dapena et al., 2020). An IOP <18 mm Hg or a reduction of 20% versus baseline was achieved in nine eyes (82%). The main complications of XEN^®^ surgery include choroidal detachment, implant dislocation and extrusion, subconjunctival haemorrhage and encapsulated blebs (Dervenis et al. 2017). The prevention of chronic hypotony has been a hallmark of the device, which utilizes an intrinsic flow‐limiting design based on the tube length and internal lumen diameter. Prospective comparative studies with larger groups of patients and longer follow‐up periods are needed to further assess the value this device (Green et al. 2018; Do et al. 2020).

### The PRESERFLO^®^ MicroShunt

The PRESERFLO^®^ MicroShunt, formerly known as the InnFocus MicroShunt, is implanted using the *ab externo* approach; however, it fulfils the MIGS criteria proposed by the American Glaucoma Society and the US FDA. The surgical technique does not require dissection of a scleral flap (as does trabeculectomy), sclerectomy or iridectomy, or flap suture placement. The bleb is placed more posteriorly and is thicker than in conventional filtering procedures (Beckers & Pinchuk 2019). The recovery time for a trabeculectomy is typically between 4 and 6 weeks, while recovery time from a MicroShunt implantation is generally around 2 weeks; the follow‐up visit is also less intensive. The implant is made of a synthetic polymer of poly(styrene‐block‐isobutylene‐block‐styrene) (SIBS) (Acosta et al. 2006; Pinchuk et al. 2017). *In vivo* studies have not reported material‐related anterior chamber reactions, bleb encapsulation, neovascularization, or inflammatory or fibrotic responses; the only reported reaction was deposition of type IV collagen near the tube (Pinchuk et al. 2017). The design of the PRESERFLO^®^ MicroShunt device is also based on the Hagen–Poiseuille equation (Arrieta et al. 2011) and is similar to that of the Xen^®^ Gel Stent. The PRESERFLO^®^ shunt tube is 8.5 mm long and 1.1 mm wide, with a 70 *µ*m inner diameter and a 350 *µ*m outer diameter. The tube has two fins that are 4.5 mm from the anterior tip and help secure the device location and prevent anterior migration. The implantation technique also utilizes MMC. An approximately 5‐mm wide fornix‐based conjunctival/Tenon's flap is created, after which a deep sub‐Tenon's pocket (6–8 mm) is formed. After marking the sclera 3 mm from the limbus, a 1‐mm deep scleral pocket is created using a triangular knife. A 25‐gauge needle is inserted into the pocket to exit the anterior chamber through the angle. The device is then advanced through the needle track in the pocket bevel into the anterior chamber, with the proximal end of the shunt extending approximately 2–3 mm into the chamber. Thereafter, the fins are secured within the scleral pocket, and flow is established by gently applying pressure on the eye or by flushing a balanced saline solution through a side‐port (Beckers & Pinchuk 2019). After checking the flow, Tenon's capsule and the conjunctiva are sutured in a watertight fashion (Pillunat et al. 2017).

The PRESERFLO^®^ MicroShunt is registered in Europe and was released in 2019 for the surgical treatment of patients with early‐to‐advanced POAG, but it has not yet been approved by the US FDA. Published evidence is limited; however, study results show that a mean IOP reduction of 30–55% from baseline can be achieved, with a substantial reduction in glaucoma medications (Canadian Agency for Drugs & Technologies in Health 2019). The longest retrospective observational study to date reports 3‐year outcomes in 23 mixed‐race patients with POAG in the Dominican Republic who received a PRESERFLO^®^ MicroShunt (14 eyes with the shunt alone, nine eyes with concomitant cataract surgery). The authors revealed that the qualified success rate (IOP ≤14 mm Hg and IOP reduction ≥20%) was 100% in the shunt alone procedure and 95% for the combined surgeries; the mean medicated IOP was reduced from 23.8 ± 5.3 to 10.7 ± 2.8 mm Hg in the group without phacoemulsification and to 10.7 ± 3.5 mm Hg in the combined surgery group. Further, the mean number of glaucoma medications was reduced from 2.4 ± 0.9 to 0.3 ± 0.7 in the former group and to 0.7 ± 1.1 in the latter group (Batlle et al. 2016).

Adverse events occur in 10–25% of cases and include hyphema (<10%), hypotony (10–16%), a shallow anterior chamber (4–13%), choroidal detachment or effusion (<9%), the device touching the iris (13%) and exposure of the Tenon's capsule (9%) (Sadruddin et al. 2019). As it is a bleb‐dependent procedure, bleb needling may be required in 2–10% of cases, usually within 9 months of follow‐up (Beckers & Pinchuk 2019). However, the device may substantially reduce IOP in contrast to most MIGS procedures, which are associated with only modest reductions in IOP; therefore, it can target patients with moderate‐to‐severe and refractory glaucoma. Only a few clinical trials with the PRESERFLO^®^ MicroShunt are currently underway (NCT01881425, NCT00772330, NCT01563237, NCT02177123; http://www.clinicaltrials.gov/).

### The CyPass Micro‐Stent

**Fig. 9:**
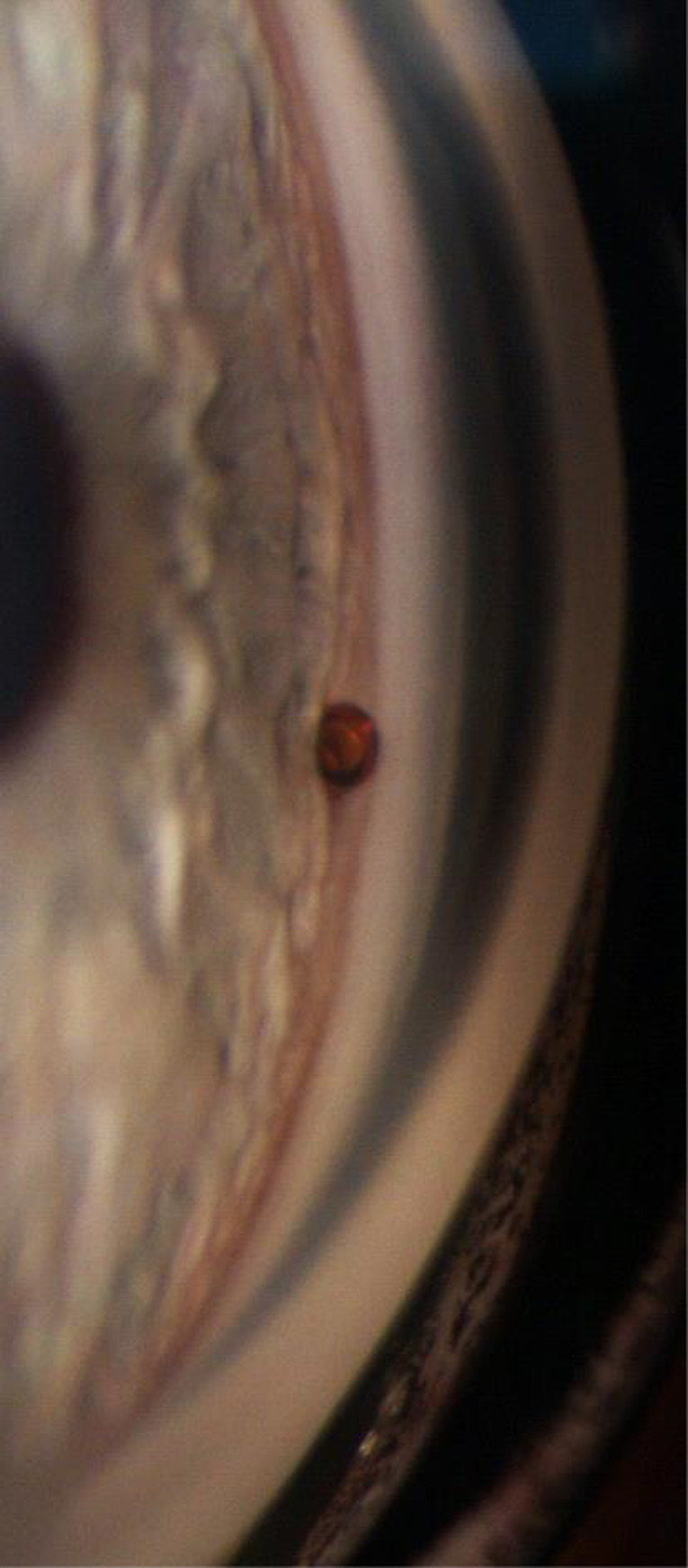
A CyPass^®^ implant in a gonioscopy

**Fig. 10:**
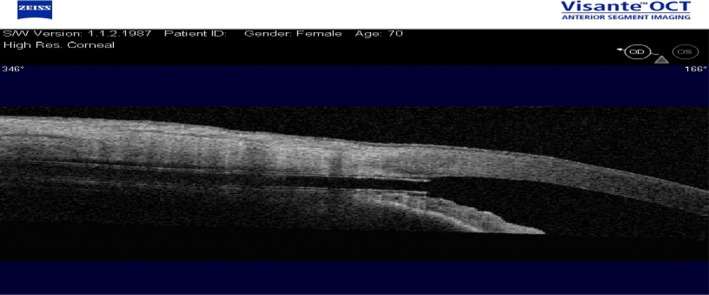
A CyPass^®^ implant observed using an OCT Visante.

**Fig. 11:**
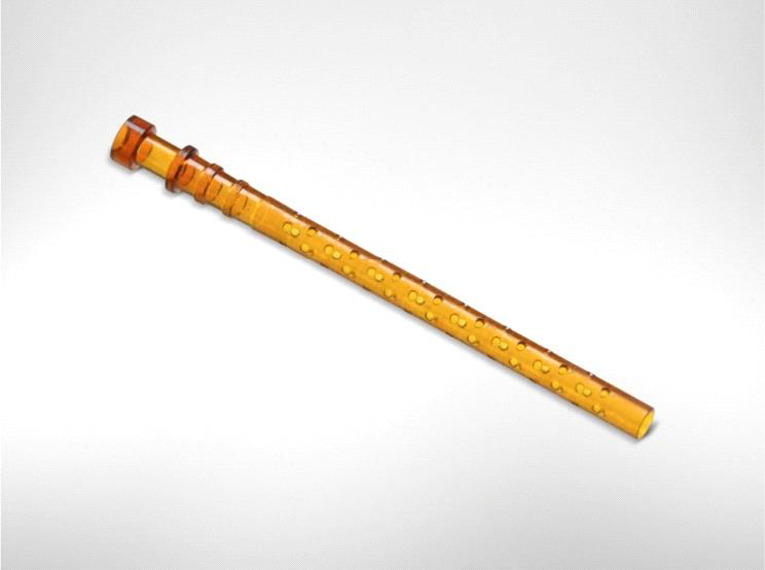
A CyPass^®^ implant.

The CyPass Micro‐Stent is implanted *ab interno* into the suprachoroidal space and was designed to achieve controlled AH outflow from the anterior chamber into the suprachoroidal space (Figs 9, 10, 11). The FDA withdrew the device from the market in 2018 (https://www.fda.gov/medical‐devices/safety‐communications/update‐potential‐eye‐damage‐alcon‐cypass‐micro‐stent‐used‐treat‐open‐angle‐glaucoma‐fda‐safety) after the detection of a dramatic rise in endothelial cell loss (ECL) among patients who received the microstent during cataract surgery, compared with patients who underwent cataract surgery alone (Compass‐XT clinical trial, NCT02700984, https://clinicaltrials.gov/). The damage likely originated from the device's positioning within the anterior chamber's angle. The ECL correlated with the number of retention rings noted on clinical examination by gonioscopy, particularly when two or more retention rings were visible.

## Discussion

The most likely point for the highest AH resistance outflow is the internal wall of SC, namely the TM. Accordingly, stent implantation through MIGS should be an effective method of treating POAG, as its IOP‐lowering effect is based on bypassing this crucial site of AH outflow resistance. This is supported by the above mentioned studies: IOP is reduced on average by 10–26% by the iStent^®^ (Spiegel et al. 2009; Seiboldet al. 2016), up to 40% by the Hydrus^®^ (Otarola et al. 2020), and 29–42% with the Xen^®^ (De Gregorio et al. 2018); the PRESERFLO^®^ MicroShunt is even better, averaging 30–55%. These studies differ widely in reported results and evidence. Notably, most involved combined procedures, rather than stand‐alone implantations.

An advantage of the mentioned techniques is *ab interno* access—access from the anterior chamber of the eye. It allows preservation of the conjunctiva and eliminates scar formation; furthermore, it enables future additional conjunctival surgery. The clear corneal incision used in cataract surgery makes it possible to expand the scope of the procedure to include MIGS without the need for additional incisions in the limbus. This has a small but significant influence on the patient's postoperative quality of life, and therefore, MIGS is often used in combination with phacoemulsification and intraocular lens implantation (Craven et al. 2012; Pillunat et al. 2017).

One limitation of all these procedures is that the postoperative IOP cannot fall below the episcleral venous pressure (EVP). The exact value of the EVP is difficult to evaluate, generally ranging from 7.6 mm Hg to 9.1 mm Hg (Zeimer et al. 1983; Sultan & Blondeau 2003) although it can be even higher in some glaucoma patients (Pillunat et al. 2017).

An open issue is the long‐term, IOP‐lowering effect of MIGS. Recent studies on human TM samples obtained intraoperatively from patients with previous implantation of a trabecular micro‐bypass stent (iStent^®^) suggest that inflammatory and fibrotic changes occur in the areas surrounding the device. These changes suggest a possible aetiology for device failure over time (Capitena Young et al., 2018). In the study with the longest follow‐up period (53.7 ± 9.3 months), the mean IOP was reduced from 19.4 ± 1.9 mm Hg preoperatively to 16.3 ± 4.2 mm Hg at the end of the observation period, indicating a 16% decrease (Arriola‐Villalobos et al. 2012). Conversely, after a 4‐year follow‐up, Fea et al. (2015) reported that the difference between the initial and final mean IOPs was not statistically significant. No SIBS obstruction have been reported for *in vivo* studies; however, accumulation of type IV collagen near the tubes has been observed (Sadruddin et al. 2019). At present, long‐term data regarding PRESERFLO^®^ MicroShunt efficacy and the other aforementioned implants are not available.

Another concern involves the effect of implants on the endothelium. The effects of CyPass and ExPress shunts and Ahmed valves on the endothelium have been studied in clinical trials (NCT02700984, https://clinicaltrials.gov/; Saheb et al. 2014; Konopińska et al. 2015). Their influence on the cornea depends on various factors, including the distance from the rear surface of the cornea, the implant material, perioperative trauma and the patient's condition before surgery (Hayashi et al. 1996). Correct positioning of the implant during surgery might reduce the associated risk. In studies of animal models, it was noted that the material of the drainage device affects the degree of cell loss (Lim 2003). The exact mechanism by which implants may damage the endothelium is not fully understood. Various theories have associated damage with an increased fluid flow around the tip of the tube, inflammatory reactions in the anterior chamber, transitory contact between the tube and the cornea or between the tube and the uvea, or immune responses evoked by the presence of a foreign body in the eye (McDermott et al. 1993). Others suggest that persistently elevated IOP directly or indirectly induces hypoxia, thereby damaging the endothelium (Ollivier et al. 2003). Fiore et al. (1989) suggested that the mechanism of endothelial damage may be associated with toxic effects of medications and preservatives contained in ophthalmic drops and with the duration of treatment; these may make the anterior chamber shallower during and after the operation or change the composition of fluids related directly to the sub‐Tenon's space. Some researchers believe that patients taking three or four concurrent antiglaucoma medications have a lower endothelium cell count compared to those taking only one or two (Lass et al. 1998). In a recent prospective study of the iStent Inject^®^, the ECL had decreased by 15% at 12 months; however, this study did not include a control group (Gillmann et al. 2020a; Gillmann et al. 2020b). Further research on ECL after MIGS implants and with long follow‐up efficacy is clearly needed.

In most cases of MIGS, implants are positioned in the nasal quadrant as, statistically, it is the site of most CCs (Grieshaber et al. 2009). Huang et al. raised the issue of the uneven distribution of CCs around SC. In their study, canalography showed a segmental distribution of CCs, which also varied among patients. This result suggests that the hypotensive success of the iStent^®^ may depend on the distribution of CCs (Huang et al. 2017; Huang et al. 2018). Individualizing and optimizing the site of micro‐bypass stent implantation by choosing the area with the largest number of CCs may become common practice.

Implant‐dependant MIGS has evolved rapidly over the past decade and is demonstrating effectiveness in reducing IOP and improving the management of glaucoma patients. They offer the desired reduction in medication usage in glaucoma patients, since not only importance is to reduce the IOP level, but as important is reducing the medication burden for patients. Further rigorous and standardized studies are needed for clinicians to better predict which patients will benefit most from each type of microdevice. Further research is also needed to determine the best approach for the appropriate primary mode of action (e.g. decrease AH production, increase trabecular or uveoscleral outflow) in cases where adjunctive medication is required.

## Supporting information


**Video S1**. iStent^®^ implantation.

**Video S2**. iStent inject^®^ implantation.

**Video S3**. iStent inject^®^ implantation 2.

**Video S4**. Hydrus® implantation.

**Video S5**. Xen^®^ implantation.
